# Opportunities and limits of the one gene approach: the ability of Atoh1 to differentiate and maintain hair cells depends on the molecular context

**DOI:** 10.3389/fncel.2015.00026

**Published:** 2015-02-05

**Authors:** Israt Jahan, Ning Pan, Bernd Fritzsch

**Affiliations:** Department of Biology, University of IowaIowa City, IA, USA

**Keywords:** Atoh1, hair cells, development, regeneration

## Abstract

*Atoh1* (*Math1*) was the first gene discovered in ear development that showed no hair cell (HC) differentiation when absent and could induce HC differentiation when misexpressed. These data implied that *Atoh1* was both necessary and sufficient for hair cell development. However, other gene mutations also result in loss of initially forming HCs, notably null mutants for *Pou4f3*, *Barhl1*, and *Gfi1*. HC development and maintenance also depend on the expression of other genes (*Sox2, Eya1, Gata3, Pax2*) and several genes have been identified that can induce HCs when misexpressed (*Jag1*) or knocked out (*Lmo4*). In the ear *Atoh1* is not only expressed in HCs but also in some supporting cells and neurons that do not differentiate into HCs. Simple removal of one gene, *Neurod1*, can de-repress Atoh1 and turns those neurons into HCs suggesting that Neurod1 blocks Atoh1 function in neurons. *Atoh1* expression in inner pillar cells may also be blocked by too many Hes/Hey factors but conversion into HCs has only partially been achieved through Hes/Hey removal. Detailed analysis of cell cycle exit confirmed an apex to base cell cycle exit progression of HCs of the organ of Corti. In contrast, *Atoh1* expression progresses from the base toward the apex with a variable delay relative to the cell cycle exit. Most HCs exit the cell cycle and are thus defined as precursors before *Atoh1* is expressed. *Atoh1* is a potent differentiation factor but can differentiate and maintain HCs only in the ear and when other factors are co-expressed. Upstream factors are essential to regulate Atoh1 level of expression duration while downstream, co-activated by other factors, will define the context of Atoh1 action. We suggest that these insights need to be taken into consideration and approaches beyond the simple Atoh1 expression need to be designed able to generate the radial and longitudinal variations in hair cell types for normal function of the organ of Corti.

## Introduction

The idea that single genes might be responsible for hair cell (HC) development and thus could be used to regenerate HCs and restore hearing was born in the late 1990s: Mice with a deletion of the Pou domain gene *Pou4f3* (aka Brn3c, Brn3.1) were completely deaf, “owing to a failure of HCs to appear in the inner ear, with subsequent loss of cochlear and vestibular ganglia” (Erkman et al., 1996). This mouse mutant derived conclusion was soon followed by data on human mutations showing that a truncating mutation of the human *POU4f3* gene is the basis of DFNA15, resulting in progressive hearing loss (Vahava et al., 1998). Subsequent work showed that HCs initially form and develop normal in *Pou4f3* mutants, but eventually die in a base to apex progression (Xiang et al., 2003; Hertzano et al., 2004). While the initial work claimed loss of all sensory neurons, later work showed that some neurons remain for 6 months in a dedifferentiated organ of Corti (OC) that shows *Atoh1-lacZ* and Myo7a positive cells (Pauley et al., 2008). The original claim of “failure of HCs to appear” was thus transformed into a rather normal initial development followed by HC death. *Pou4f3* is now recognized as a maintenance factor for HCs, like *Gfi1* and *Barhl1* (Li et al., 2002; Hertzano et al., 2004) that is expressed in adult HCs through complex regulation, including possibly the bHLH gene Atoh1 (Ahmed et al., 2012; Masuda et al., 2012).

Why is this background information on *Pou4f3* relevant for the discussion of the role of *Atoh1* (aka *Math1*) for HC differentiation and maintenance? In the following we will explore that *Atoh1* has much in common with *Pou4f3* in terms of claims raised as a gene that is “necessary and sufficient” for HC differentiation (Chen et al., 2002; Giraldez and Fritzsch, 2007; Groves et al., 2013). In contrast to this claim, the millions of neurons outside the ear expressing Atoh1 (Mulvaney and Dabdoub, 2012) never turn into HCs, suggesting that Atoh1 is not sufficient to induce HCs everywhere where Atoh1 is expressed. Only the molecularly unclear context of certain cells of the ear allows Atoh1 to drive HC differentiation and maintenance. Even in the ear, Atoh1 is expressed in many cells (Matei et al., 2005) that require additional manipulations to turn into HCs (Jahan et al., 2010), indicating that expression of Atoh1 in the ear does not guarantee differentiation of all cells into HCs. As with Pou4f3, it appears that Atoh1 absence is compatible with some cellular differentiation, indicating that Atoh1 is not defining HCs but is differentiating them (Jahan et al., 2012). The delayed and profound loss of HCs in a “self-terminating” Atoh1 system (Pan et al., 2012) and hypomorphic Atoh1 mutant (Sheykholeslami et al., 2013) suggests an essential role in maintenance, possibly including adult expression of Pou4f3 (Masuda et al., 2012). Consistent with Atoh1 being an essential differentiation and maintenance factor for HC is the fact that overexpression can rescue HCs (Yang et al., 2012). Like Pou4f3, Atoh1 is necessary to differentiate and maintain HCs. It remains to be shown whether forced expression of Atoh1 (Kelly et al., 2012) can differentiate HCs when certain factors are absent (Zou et al., 2004; Kiernan et al., 2005; Bouchard et al., 2010; Ahmed et al., 2012; Duncan and Fritzsch, 2013; Schimmang, 2013) that define the context for Atoh1 action in the ear thus providing the competency to respond to Atoh1 protein. Below we explore some issues related to Atoh1 function that remain underexplored in many contemporary reviews and propose novel strategies to maintain HCs.

## Expression of Atoh1 outside the ear does not lead to HC differentiation

*Atoh1* was isolated from cerebellar granule cells, the largest population of neurons in the human brain, amounting to over 60 billion neurons (Ben-Arie et al., 1997; Herculano-Houzel, 2009). Atoh1 is expressed in the proliferative precursor population of the external granule cell layer where it is needed to generate the billions of granule cells (Pan et al., 2009). *Atoh1* is also essential for medulloblastoma progression and *Atoh1* removal reduces the progression of this childhood tumor (Flora et al., 2009). In contrast to this expression of *Atoh1* in proliferating precursors in the CNS, the expression of *Atoh1* in the mouse cochlea is predominantly in post-mitotic HCs, with a possible overlap of *Atoh1* expression and cell cycle exit in the basal turn HCs (Ruben, 1967; Matei et al., 2005; Lee et al., 2006). A pulse-chase experiment using BrdU or EdU labeling followed by *in situ* hybridization for *Atoh1* around E14 is needed to verify this suggestion of possible *Atoh1* expression in proliferating HC precursors. In the apex there is no expression of Atoh1 prior to cell cycle exit, indicating that HC precursor specification and cell cycle exit is independent of *Atoh1* (Jahan et al., 2013; Kopecky et al., 2013). Both premature expression of Atoh1 in *Neurod1* null mutants (Jahan et al., 2010) or delayed expression of *Atoh1* in *Lmx1a* null mice (Nichols et al., 2008) results in aberrant development of HCs, implying that onset and level of expression of *Atoh1* is tightly regulated to ensure normal differentiation of the right HC type at the right place (Jahan et al., 2013). Importantly, forced expression of Atoh1 can in postnatal mice induce supporting cell conversion (Liu et al., 2014a) and induces proliferation (Kelly et al., 2012), showing that under these forced conditions Atoh1 exerts functions beyond its tightly regulated function in the embryonic ear. In summary, one of the conditions in which *Atoh1* expression in the ear differs from other systems is its expression presumably exclusively in post-mitotic undifferentiated HC precursors whereas in other developing systems *Atoh1* is primarily expressed in proliferating precursors.

## Upstream and downstream interactions of Atoh1

**Figure 1:**
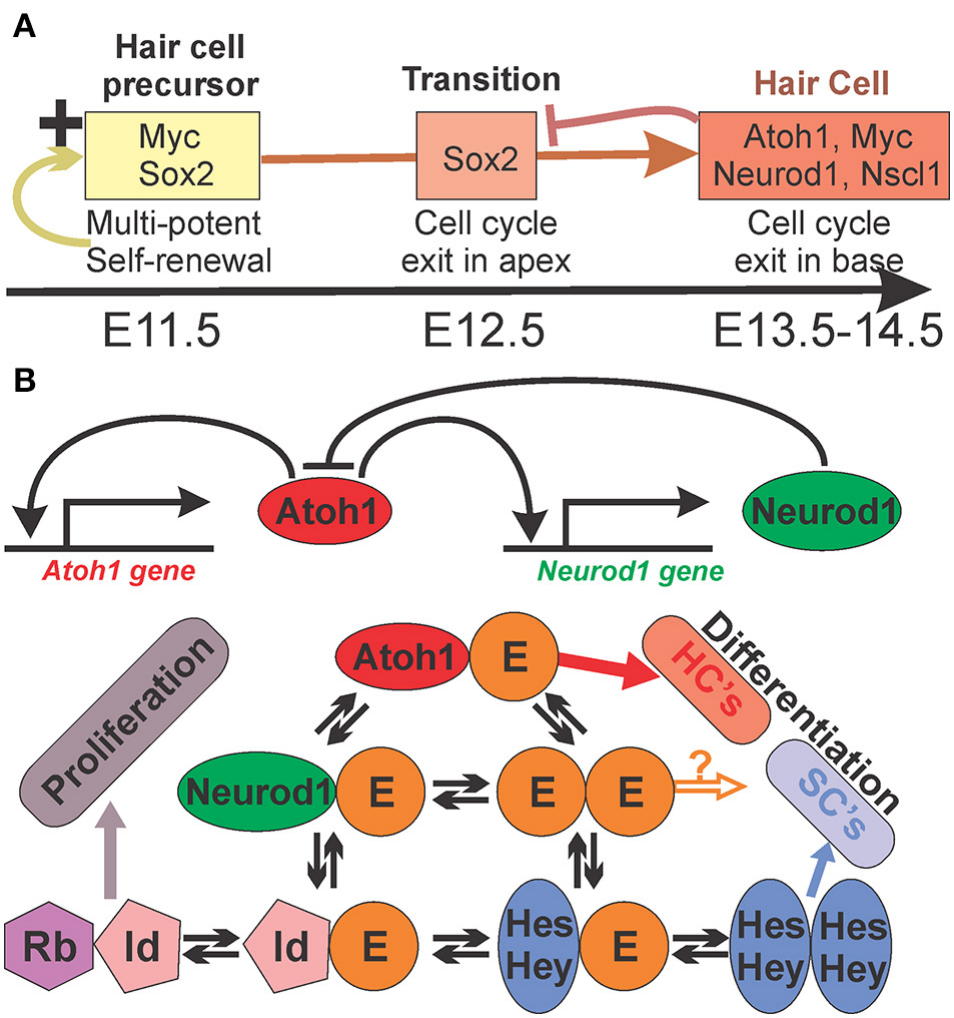
**The interactions of Sox and bHLH genes in HC differentiation in mice**. Experimental data indicate a complex interaction of Sox and bHLH genes in the progression of HC fate commitment and differentiation. **(A)** Sox2 and Myc genes act downstream of Eya1/Six1 and are essential for proliferation of HC precursor cells to ensure self-renewal of precursors but also commitment to the HC lineage. These precursor genes are turned off in the neurosensory lineage and bHLH genes are activated that antagonizes Sox2. **(B)** bHLH TFs can form complex interactions in a given cell that can undergo periodic changes in expression levels and their signal can undergo context dependent variation between gene expression and suppression. Data in mice and flies suggest that all proneural TFs compete for the E-proteins (Tcf3,4,12) to form heterodimers for proper binding. Thus, the level of all proneuronal bHLH TFs (here Atoh1 and Neurod1) and available E-proteins as well as their binding preference will determine how much signaling of heterodimers will occur. Importantly, E-proteins can also interact with Hes/Hey factors and the inhibitors of DNA binding (Ids), limiting availability of E-proteins for heterodimerization of proneuronal protein, proportionally to the affinity and concentration of all these interactive partners. In essence, the binding properties and frequency of the binding partners will determine whether a cell is differentiating as a neuron/HC, a supporting/glial cell, or is continuing proliferation as a prosensory precursor. HC, hair cell; SC, supporting cell. Modified after (Fritzsch et al., 2015a).

Before Atoh1 can differentiate post-mitotic HC precursors into HCs, the HC precursors have to be specified in the right place and have to receive a signal to exit the cell cycle. Numerous TFs have been identified that are expressed prior to *Atoh1* and affect HC differentiation. For example, *Sox2* hypomorphic mice (Kiernan et al., 2005), *Pax2* null mice (Bouchard et al., 2010), *Eya1* null mice (Zou et al., 2004), and *Gata3* conditional null mice (Duncan and Fritzsch, 2013) all show no differentiation of HCs in the cochlea duct but may show variable development of some vestibular HCs, suggesting a unique combinatorial requirement of these genes for cochlear HC development. Misexpression of *Jag1* (Pan et al., 2010) or *Sox2* (Pan et al., 2013) as well as loss of *Lmo4* (Deng et al., 2014) can induce ectopic formation of HCs. In particular work on *Eya1/Six1* showed that *Atoh1* is but an essential link in a succession of decision making steps (Ahmed et al., 2012) toward HC differentiation (Figure 1) with unknown regulatory complexity.

*Atoh1* regulates the expression of hundreds of downstream genes (Klisch et al., 2011). Some of these genes are TFs that in turn regulate expression of several hundred downstream genes. One of the TFs that are regulated by Atoh1, is *Neurod1*. Atoh1 is in a positive autoregulatory loop whereby Atoh1 stimulates its own expression through an enhancer sequence (Figure 1). Such loops are typically counterbalanced by negative feedback to ensure upper limits of expression. Neurod1 is part of this negative feedback loop and controls the level of Atoh1 expression in developing systems such as the cerebellum (Pan et al., 2009), the intestine (Itkin-Ansari et al., 2005), and the ear (Jahan et al., 2010, 2013). Absence of Neurod1 causes prolonged expression of *Atoh1* in precursor cells (external granule cell layer) of the cerebellum that are unable to migrate and differentiate and eventually die (Pan et al., 2009). In the ear, absence of *Neurod1* causes transformation of sensory neurons into HCs through disinhibition of a transient Atoh1 in neurons (Jahan et al., 2010) and disruption of the patterning of the OC by altering the HC and supporting cell types (Jahan et al., 2013). Some regulation of *Atoh1* is also reported in mutants of Hes1/5 (Zine et al., 2001, 2014) and Hey1/2 (Benito-Gonzalez and Doetzlhofer, 2014) but results only in additional formation of HCs outside the OC with limited effects on the patterning of HCs and supporting cells in the OC. Atoh1 is not only regulating the expression of downstream genes but also suppresses upstream genes such as *Sox2* (Figure 1). In fact, downregulation of Sox2 appears to be a crucial step for the transition from HC precursors to differentiated HCs (Dabdoub et al., 2008) in agreement with many other differentiating neurosensory system (Reiprich and Wegner, 2014).

Combined, these data show that the early implications of *Atoh1* as the “sole” factor necessary and sufficient to make HCs have to be adjusted to accommodate the emerging concept of Atoh1 integration into a gene network that allows a coordinated transition from the placodal stage to the fully differentiated HC (Ahmed et al., 2012). Arguably, Atoh1 is enabling a very essential step in this progression toward a HC, but is apparently not needed for precursors to exit the cell cycle and to initiate HC differentiation (Jahan et al., 2012). However, Atoh1 is a key to HC differentiation (Kelly et al., 2012) and its continued expression may be essential to maintain differentiated HCs through expression of other genes such as Gfi1, Pou4f3, and Barhl1 (Masuda et al., 2012).

## Cell cycle exit and Atoh1 expression

Proliferating neurosensory precursor cells are characterized by the expression of multiple transcription factors (TFs) (Ono et al., 2014) and manipulating cell cycle regulation can result in increased (Mantela et al., 2005; Schimmang and Pirvola, 2013) or decreased HCs (Kopecky et al., 2011, 2013). Together these factors ensure that proliferating HC precursors retain a neurosensory determination but continue proliferation to generate more neurosensory cells, under certain conditions and in certain species as stem cells throughout life, like in the olfactory system (Gokoffski et al., 2011). Nearly ubiquitous in these stem cells is the expression of Sry-box gene Sox2 (Reiprich and Wegner, 2014) and several Helix-loop-Helix (HLH) genes (Figure 1), in particular *Hes*, N-*Myc* and *ID* genes, but also some proneural basic Helix-Loop-Helix (bHLH genes) such as *Ascl1*, *Neurog1* and, rarely, *Atoh1* (Ma et al., 1996, 1998; Imayoshi and Kageyama, 2014). Sox genes and bHLH genes are each engaged in a complicated interaction with members of their own class of genes within a given precursor (Fritzsch et al., 2006; Imayoshi and Kageyama, 2014; Reiprich and Wegner, 2014) but also show cell-cell interactions through Delta-Notch mediated regulation of bHLH genes between cells (Benito-Gonzalez and Doetzlhofer, 2014). In particular, the intracellular interactions established through intrinsic and extrinsic signal mediated fluctuation of expression levels is the basis for a coordinated transition between precursors and differentiated cells (Figure 1). How HC precursors are specified in the right topology of the OC, how the cell cycle exit of HC precursors is regulated and exactly when precursors are committed to HC differentiation by which molecular means remains an open question despite recent insights into the regulation (Ahmed et al., 2012; Masuda et al., 2012). Among bHLH genes, Myc genes are playing a major role in regulating the numbers of HCs (Domínguez-Frutos et al., 2011; Kopecky et al., 2011) but are later also expressed in adult HCs where they play no discernable function (Kopecky et al., 2012). We will here explore only the role of proneural bHLH genes in this process, also other TFs undoubtedly play a role in HC specification and proliferation (Kiernan et al., 2005; Dabdoub et al., 2008; Rocha-Sanchez et al., 2011; Ahmed et al., 2012; Schimmang and Pirvola, 2013).

## Losing Atoh1 at different stages results in different effects

In the original paper describing absence of HC differentiation in Atoh1 null mutant mice, some supporting cells stain for the LacZ used to replace Atoh1 (Bermingham et al., 1999). A follow up study using an Atoh1 enhancer element to drive fluorescent GFP (Chen et al., 2002) showed that Atoh1 is only expressed in post-mitotic cells to drive their differentiation. In addition, degenerative cells were found in the OC of Atoh1 null mice, suggesting that the primordial HCs form independently of Atoh1 but degenerate without Atoh1. Both papers indicated one major difference: Atoh1-LacZ expression in supporting cells of Atoh1 null mutants whereas no such misexpression was reported using GFP. A subsequent paper using the LacZ insertion (Woods et al., 2004) claimed an initial widespread expression at E13.5. This paper did not correlate the apparent absence of Atoh1 expression in the apex with the HC cycle exit known to start in the apex (Ruben, 1967; Fritzsch and Nichols, 1993).

Using the same LacZ knockin model as previous papers (Bermingham et al., 1999; Woods et al., 2004), a follow up paper on homozygotic Atoh1-LacZ mice showed continued presence of a single row of undifferentiated LacZ positive cells (Fritzsch et al., 2005) which were spared by the otherwise prevalent apoptosis of most HC precursors (Chen et al., 2002). Subsequent work demonstrated that fluorescent GFP marker (Chen et al., 2002) appeared in nearly every inner pillar cell (Matei et al., 2005; Fritzsch et al., 2015b). Furthermore, a novel mouse line using the same enhancer element to drive Cre showed similar expression of Atoh1 in many inner pillar cells (Matei et al., 2005). These data implied, but did not proof beyond doubt that Atoh1 was expressed in inner pillar cells and inferred that the remaining Atoh1-LacZ positive cells in mutants were indeed supporting cells (possibly inner pillar cells) as originally claimed (Bermingham et al., 1999). Further work using a conditional approach to eliminate Atoh1 resulted in nearly identical data, implying that the surviving cells in the absence of Atoh1 might indeed be inner pillar cells in the OC (Pan et al., 2011). Additional work has meanwhile confirmed with different techniques that Atoh1 is indeed prominently expressed in inner pillar cells (Driver et al., 2013). *Atoh1* expression in inner pillar cells may be counterbalanced by Hes and Hey factors (Doetzlhofer et al., 2009) and a subsequent paper showed occasional conversion of inner pillar cells to HCs (Benito-Gonzalez and Doetzlhofer, 2014). Atoh1 expression has also been reported in delaminating sensory neurons (Matei et al., 2005) and elimination of *Neurod1* suffices to turn some neurons into HCs expressing Atoh1 and Myo7a (Jahan et al., 2010). Combined, these data suggest that Atoh1 expression alone does not suffice to turn just any cell in the ear into a HC as co-expressed factors may inhibit this. At least inner pillar cells may be able to survive without Atoh1 protein while maintaining LacZ expression of the Atoh1 locus (Matei et al., 2005; Driver et al., 2013) and are not transformed to HCs even under forced ubiquitous expression of Atoh1 (Kelly et al., 2012).

More recent data provide yet a more complicated picture of lack of Atoh1 expression on HC and OC differentiation. Using an Atoh1 enhancer to drive Cre that activates the Cre only upon presence of Atoh1 protein combined with floxed *Atoh1* generates a “self-terminating” system that results in loss of *Atoh1* after a transient presence of Atoh1 protein (Pan et al., 2012). The level of Atoh1 protein depends on the speed with which the Cre can excise the floxed *Atoh1* and how long residual Atoh1 protein remains in the cell. Thus, while all cells will see recombination of the LoxP flanked *Atoh1*, this varies between HCs and thus results in different delay lines of HC precursor apoptosis (Pan et al., 2012). While many HC precursors die rapidly, others survive for several days. Moreover, stretches of the first row of outer HCs survive adjacent to well differentiated inner pillar cells indicating an unusual difference in susceptibility between inner and outer HCs as well as within HC rows in a base to apical gradient. This conclusion is also supported by transgenic knockin mouse where *Atoh1* is replaced by *Neurog1* (Jahan et al., 2012) which shows that some HC precursors can survive without ever expressing *Atoh1*. A recently available hypomorph mutant of *Atoh1* shows a somewhat similar picture of longitudinal and a less clear radial HC loss (Sheykholeslami et al., 2013) indicating that Atoh1 needs to be present at a critical level to assure long term HC viability.

Data using inducible Cre expression have complicated this picture even further by showing a rapid and complete loss of all HCs when Cre is induced at different stages of late development (Cai et al., 2013; Chonko et al., 2013). Some claims about abortive transdifferentiation of supporting cells into HCs (Cai et al., 2013) need to be considered in the context of Atoh1 expression in one specific type of supporting cell, the inner pillar cell (Matei et al., 2005; Driver et al., 2013; Fritzsch et al., 2015b). Despite these minor discrepancies, all papers confirm earlier work and demonstrate that Atoh1 expression is needed to mature and maintain HCs.

In summary, Atoh1 is, much like Pou4f3, a critical factor for HC differentiation and long term maintenance. Atoh1 is involved in regulating Pou4f3 whereas and its long term expression may be dependent on Atoh1 expression. Further work combining the recently reported hypomorphic allele (Sheykholeslami et al., 2013) with conditional deletion of a floxed Atoh1 allele (Pan et al., 2012) could detail how level of Atoh1 expression and duration combine for normal HC maturation and maintenance.

## Summary and outlook

Why is it important to go beyond the idea of “necessary and sufficient” for Atoh1 function in the ear? First, while unregulated expression of Atoh1 can convert most ear cells into hair cells (Kelly et al., 2012), nobody has been able to regenerate the two types of HCs that are essential for normal OC function in the right proportion and the right distribution to ensure function (Beurg et al., 2014). In fact, our limited insights into the molecular basis of this crucial aspect of HC differentiation (Jahan et al., 2013) are not profound enough to regenerate the right type of HC (Liu et al., 2014b) to ensure normal function. Defining the molecular context needed for HC type specific differentiation in conjunction with defined levels of Atoh1 expression (Jahan et al., 2013) and controlled changes of Atoh1 expression over time (Ahmed et al., 2012) will be needed to move forward.

Second, most HCs generated with Atoh1 treatment alone have limited long term viability. In part this may relate to the progressive loss of Atoh1 in these experiments that may needed to maintain long term Pou4f3 expression (Masuda et al., 2012), but in part it may also relate to an unstable transformation into HC that requires recapitulating the specification sequence of HCs precursors and their differentiation. Such critical steps might include expression of additional factors prior to and in addition to Atoh1 or the prolonged expression of critical levels of Atoh1. Human hearing loss may show partial dedifferentiation of the OC with profound local differences comparable to experimental animals (Taylor et al., 2012). A “one size fits all” approach to such heterogeneity may result in incomplete restoration.

Finally, while the single gene approach to HC regeneration has been extremely influential to catapult much research forward, it is now time to reflect why this approach has not lived up to its promise. We therefore suggest more complex procedures that recapitulate steps in development of the OC in addition to Atoh1. For example, expressing Eya1, Pax2, Sox2, Jag1, Foxg1, Neurod1, Neurog1, and Gata3 prior to Atoh1 expression may “prime” remaining cells of the OC to respond to Atoh1. Alternatively, combining Atoh1 with downstream essential genes for HC maintenance that are only partially regulated by Atoh1 (Ahmed et al., 2012), such as Pou4f3, could define the context for HC differentiation. Moreover, using transient expression of Atoh1 in already differentiated HCs might prolong their viability (Yang et al., 2012), possibly long enough to sidestep the need for OC regeneration in elderly people suffering from early stages of neurosensory hearing loss. Given the projected massive occurrence of hearing loss in the next 25 years, ideas revolving around maintenance of HCs using Atoh1 alone might provide more-short term benefit compared to currently impossible reconstitution of the OC after long term HC loss. Given the ability of Atoh1 to transdifferentiate supporting cells in certain conditions (Liu et al., 2014a), it might be necessary to replace Atoh1 by other bHLH genes that can accomplish long term maintenance of HCs without risk of transforming supporting cells into HCs. We are currently working on such approaches using novel mouse models to differentiate HCs in the absence or at most transient presence of Atoh1.

### Conflict of interest statement

The authors declare that the research was conducted in the absence of any commercial or financial relationships that could be construed as a potential conflict of interest.
